# The Common Bean *V* Gene Encodes Flavonoid 3′5′ Hydroxylase: A Major Mutational Target for Flavonoid Diversity in Angiosperms

**DOI:** 10.3389/fpls.2022.869582

**Published:** 2022-03-31

**Authors:** Phillip E. McClean, Rian Lee, Kevin Howe, Caroline Osborne, Jane Grimwood, Shawn Levy, Amanda Peters Haugrud, Chris Plott, Melanie Robinson, Ryan M. Skiba, Tabassum Tanha, Mariam Zamani, Theodore W. Thannhauser, Raymond P. Glahn, Jeremy Schmutz, Juan M. Osorno, Phillip N. Miklas

**Affiliations:** ^1^Department of Plant Sciences, North Dakota State University, Fargo, ND, United States; ^2^Genomics, Phenomics, and Bioinformatic Program, North Dakota State University, Fargo, ND, United States; ^3^USDA-ARS, Robert W. Holley Center for Agriculture and Health, Cornell University, Ithaca, NY, United States; ^4^Genome Sequencing Center, HudsonAlpha Institute for Biotechnology, Huntsville, AL, United States; ^5^USDA-ARS, Grain Legumes Genetics and Physiology Research Unit, Prosser, WA, United States

**Keywords:** common bean, F3′5′ hydroxylase, flavonoid biosynthesis, flavonoid composition, legumes, mutational targets, protein modeling, recombination mapping

## Abstract

The classic *V* (violet, purple) gene of common bean (*Phaseolus vulgaris*) functions in a complex genetic network that controls seed coat and flower color and flavonoid content. *V* was cloned to understand its role in the network and the evolution of its orthologs in the Viridiplantae. *V* mapped genetically to a narrow interval on chromosome Pv06. A candidate gene was selected based on flavonoid analysis and confirmed by recombinational mapping. Protein and domain modeling determined *V* encodes flavonoid 3′5′ hydroxylase (F3′5′H), a P450 enzyme required for the expression of dihydromyricetin-derived flavonoids in the flavonoid pathway. Eight recessive haplotypes, defined by mutations of key functional domains required for P450 activities, evolved independently in the two bean gene pools from a common ancestral gene. *V* homologs were identified in Viridiplantae orders by functional domain searches. A phylogenetic analysis determined F3′5′H first appeared in the Streptophyta and is present in only 41% of Angiosperm reference genomes. The evolutionarily related flavonoid pathway gene flavonoid 3′ hydroxylase (F3′H) is found nearly universally in all Angiosperms. F3′H may be conserved because of its role in abiotic stress, while F3′5′H evolved as a major target gene for the evolution of flower and seed coat color in plants.

## Introduction

Common bean (*Phaseolus vulgaris*) is the most consumed food legume in the world and is widely considered a highly nutritious crop. The societal impact of the crop is greatest for smallholder farmers that rely on it as a family food and a source of cash. Common bean seeds are painted with a wide array of colors and patterns. The colors, as for all members of the plant kingdom, are determined by their flavonoid composition and concentration (Lin et al., 2008; Iwashina, 2015; Madrera and Valles, 2020). These colors and patterns define the many bean market classes regionally preferred by peoples throughout the world. From an economic perspective, large and smallholder growers rely on stable expression of the seed traits for marketability of their crop. From a health perspective, common bean is widely appreciated as a healthy food partially because of its flavonoid content. And from an eco-physiological perspective, flavonoids are associated with abiotic stress resistance (Šamec et al., 2021). As such, a long-term goal of bean genetics is to define the molecular nature of the genes controlling seed color.

The color and pattern of bean seeds and flowers are controlled by a detailed genetic network (Bassett, 2007). *P* is the master regulator of the network, and a dominant allele is required for color expression in the plant. *G* (Lundberg and Åkerman, 1917), *B* (Johannsen, 1909), and *V* (Johannsen, 1909) interact in various allelic combinations to color the seed from yellow to black (Lamprecht, 1932; Prakken, 1970). Since *G*, *B*, and *V*, were associated with the expression of a particular flavonoid pigment (Beninger and Hosfield, 2003), it was hypothesized that one or all of these genes may encode a flavonoid biosynthetic enzyme. *V* also has pleiotropic effects on flower color where the dominant *V* allele expresses purple flower color, and several recessive alleles control pink or white flowers (Lamprecht, 1936). The eight *GBV* allelic combinations are further modified by recessive *rk* alleles (Gloyer, 1928; Smith, 1939; Bassett and Miklas, 2003), which add light red tinges to the seed, or the dominant *R* allele, that imparts darker red colors (Smith, 1939; Bassett, 2007). Two genes, *M* (Shull, 1908) and *S* (Tjebbes and Kooiman, 1919), linked in the complex *C* locus (Prakken, 1974), control mottled and striped seed coat patterns, respectively. Other partial seed coat patterns are only expressed in the presence of the recessive *t* allele (Shaw and Norton, 1918) and its interaction with the *Z*, *Bip*, *J*, and *Fib* genes (see Table 8.21, Bassett, 2007). Genetic mapping data (McClean et al., 2002) and the release of the reference assembly and annotation of the bean genome (Schmutz et al., 2014) were combined to provide an approximate physical location of many of these genes and a starting point for the discovery of the molecular nature of each gene (Reinprecht et al., 2013).

**Figure 1:**
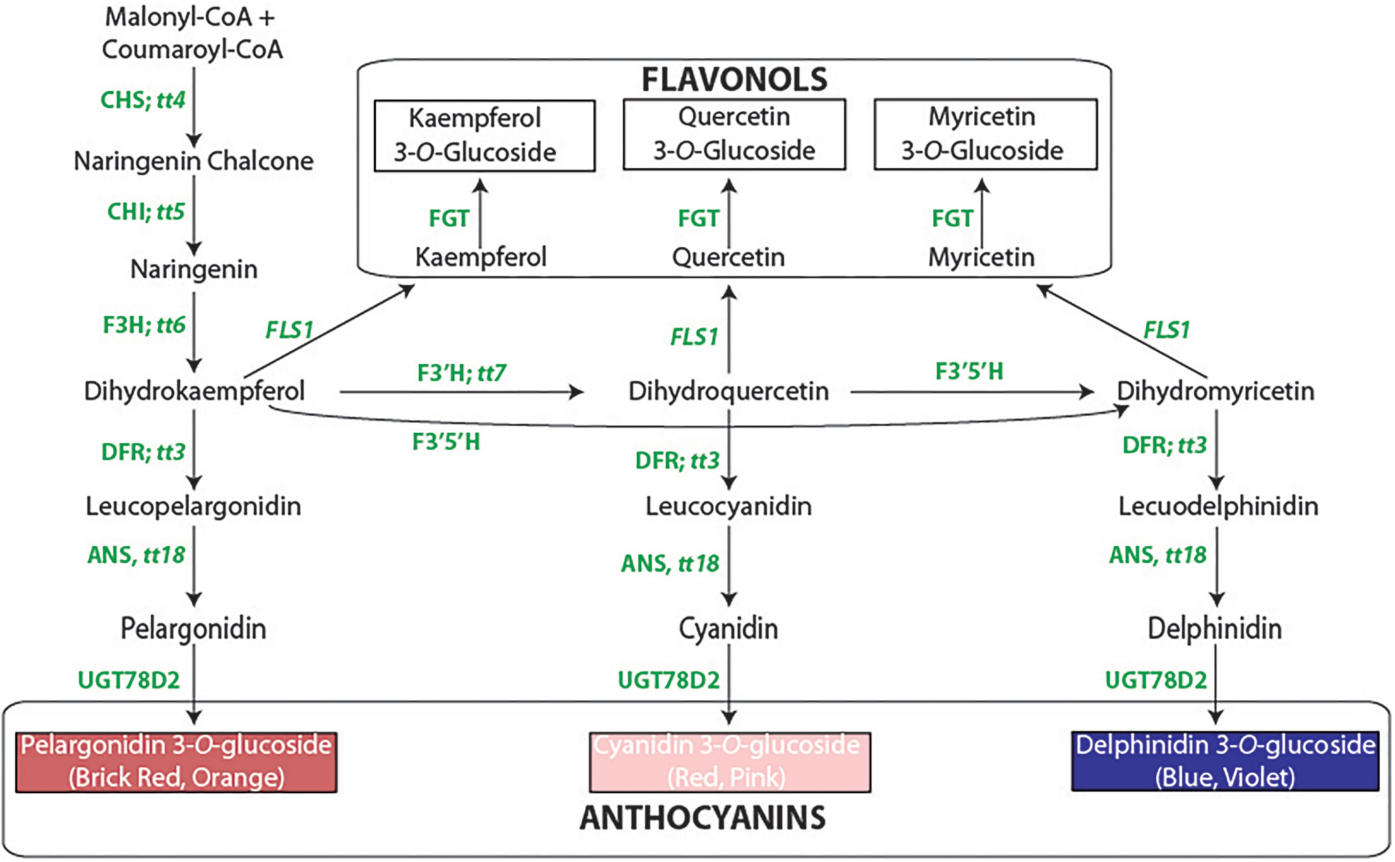
The flavonoid biosynthetic pathway. The enzyme abbreviations [followed by the *Arabidopsis* gene symbols, beginning with *tt* = transparent testa]: CHS, chalcone synthase; CHI, chalcone isomerase; F3H, flavanone 3-hydroxylase; F3′H, flavonoid 3′-hydroxylase; F3′5′H, flavonoid 3′5′-hydroxylase; DFR, dihydroflavonol 4-reductase; ANS, anthocyanidin synthase; UGT78D2, flavonol-3-*O*-glucosyltansferase; FLS, flavonol synthase. The methylated derivatives of cyanidin (peonidin) and delphinidin (petunidin and malvidin) are not shown.

The detailed genetic background suggests that some, if not all, of the color/pattern genes are either enzymatic or regulatory components of the flavonoid pathway (Figure 1). The cloning of *P* provided initial support for this concept (McClean et al., 2018). It was determined that *P* encodes a βHLH regulatory protein that is an ortholog of genes that are part of the MBW (MYB-βHLH-WD40) ternary complex that activates the late biosynthetic proteins required to produce anthocyanins and proanthocyanins in other species (see Lloyd et al., 2017 for a historical review of the MBW complex). Most recessive *p* alleles eliminate flavonoids rendering seeds and flowers white. One allele, *P*^SD^ (Islam et al., 2020), is unique. It allows normal seed color development, but the typical post-harvest seed darkening observed with the *P* allele is greatly delayed when *P*^SD^ is present. The delayed darkening is associated with a significant reduction in the expression of dihydroflavonol reductase and anthocyanin reductase (Islam et al., 2020) and the synthesis of procyanidins (Wiesinger et al., 2021) that are precursors of proanthocyanins whose oxidation darkens the seed. Since procyanidins are inhibitors of iron uptake (Hart et al., 2015, 2017), iron is more readily available from seeds of *P*^SD^. This is one example that integrates the seed color genetic network with the flavonoid pathway and the health-promoting effects of beans. Understanding the molecular nature of other genes of the network will better inform efforts to improve bean nutrition.

The focus here is on the *V* gene that differentially affects color in seeds and flowers. In plants, loss of color in an organ is generally caused by mutations in regulatory genes, while a change in hue results from an enzymatic gene mutation (Streisfeld and Rausher, 2011). Like recessive *p* alleles, *V* mutants greatly reduce or eliminate flower pigmentation. In contrast, seed color is eliminated with most mutant *p* alleles, while seed color is expressed in mutant *v* genotypes. Therefore, cloning *V* is necessary to determine if it is a regulatory gene in the flavonoid pathway similar to a MYB repressor that eliminates floral color in *Iochroma* (Gates et al., 2018), or an enzymatic factor, possibly in the flavonoid pathway. The discovery that common bean ancestors evolved into the wild Middle American and Andean gene pools ~111 Kya (Mamidi et al., 2013), and that each pool was subsequently domesticated independently (Gepts et al., 1986), must be considered for any complete molecular analysis of any common bean phenotype. Thus, cloning *V* will also enable an assessment of the molecular features that distinguish the dominant and recessive alleles in the two gene pools. This will also address the question of whether recessive *v* alleles evolved independently in the two gene pools, or whether gene pool-specific dominant *V* alleles evolved from an ancestral allele and subsequently evolved into gene pool-specific recessive *v* alleles.

To address these questions, it was necessary to discover the molecular nature of *V*. This required biochemical, molecular, and structural experiments to definitively clone *V*. Early chemical analyses of seed coats by Feenstra (1960) and Beninger and Hosfield (2003) found that genotypes carrying the recessive *v* allele did not produce delphinidin anthocyanins, molecules that require the action of flavonoid 3′5′ hydroxylase (F3′5′H). F3′5′H is a member of the cytochrome P450 (CYP) CYP75A subfamily (Toguri et al., 1993). Here, we describe biochemical analysis of introgression lines, with a shared background and different recessive *v* alleles, and found that dihydromyricetin-related compounds, including delphinidin, were greatly reduced in seeds and flowers with recessive *v* alleles. We physically mapped *V* near a F3′5′H gene model, a result also reported recently using RIL mapping by García-Fernández et al. (2021). Our sequencing of natural variants and a haplotype reconstruction conclusively shows that *V* indeed encodes F3′5′H. The mutational landscape of *V* was considered by determining the evolutionary origins of the dominant purple and recessive white alleles in the two gene pools and how they might be related to a pink flower mutation. Subsequently, phylogenetic and protein structural analyses were performed to consider the breadth of functional orthologs of *V* across the Viridiplantae and the evolution of F3′5′H relative to its related P450 protein flavonoid 3′ hydroxylase (F3′H).

## Materials and Methods

### Plant Material

Dr. Mark Bassett, University of Florida, developed a large set of backcross introgression lines using 5-593 (PI 608674; black seed, purple flower) as the recurrent parent and donor lines with specific alleles that affect flower and seed coat color and pattern (Bassett, 2007). 5-593 carries the dominant allele for all but two of the genes. Its genotype for these genes is: *T P* [*C r*] *J G B V Rk Gy sal*. Introgression and donor lines used in this research are listed in Table 1. The nomenclature for the lines begins with the introgressed allele, followed by the level of backcrossing to 5-593. For example, *g v* BC_3_ 5-593 means the recessive *g* and *v* alleles were introgressed to the BC_3_ generation with 5-93 as the recurrent parent. Other genotypes used for resequencing are described in Supplemenatry Table S1. An F_2_ population [*g v* BC_3_ 5-593 (brown seed, white flowers) x Black Magic black seed, purple flower; *n* = 120] was developed and scored for flower color.

**Table 1:** Genetic resources used in *V* and flavonoid 3′5′ hydroxylase mapping and sequencing experiments.

Genetic resource type	NPGS accession	Genotype name	*V* allele introgression donor	*V* allele	Phenotype
Recurrent parent	PI 608674	5–593		*V*	Black seed; purple flower
Introgression line	PI 608684	*g v* BC_3_ 5–593	ICA-Calima	*v*	Gray-brown seed; white flowers
Introgression line	PI 608680	*v*^lae^ BC_3_ 5–593	V0491	v^lae^	Mineral brown seed; pink flowers
Introgression line	PI 608679	*v* BC_3_ 5–593	M0056	*v*	Mineral brown seed; pink flowers^a^
Allele donor	PI 642948	ICA-Calima		*v*	Red over cream seed; white flowers
Allele donor	PI 527745	V0491		*v* ^lae^	Mineral brown seed; pink flowers
Allele donor	PI 527830	M0056		*v*	Mineral brown seed; pink flowers^a^
Andean: Purple flower	W6 9,664	Ivajlovgrad 2		*V*	Black seed; purple flower
Andean: Purple flower	PI 146751	Black Wonder		*V*	Black seed; purple flower
Andean: Purple flower	PI 638810	RH No. 6		*V*	Black seed; purple flower

*NPGS (U.S. National Plant Germplasm System:* *https://npgsweb.ars-grin.gov/**)*.

a *Pink flowers appear because of dominant Sal allele not V allele*.

### LC–MS Quantification of Polyphenols

See Supplementary Table S2 for chemical sources.

Seed coat powders were prepared as described previously (Hart et al., 2020). Freeze-dried flower samples were ground into powder. The powders were weighed and initially solubilized with a 50% solution of methanol in water and dried using a Labconco vacuum concentrator (Kansas City, MO). Samples were reconstituted with 2 ml (flower) and 3 ml (seed coat) of 50% dimethyl sulfoxide solution in water. Standard curves were also prepared in 50% DMSO.

Samples were analyzed *via* reversed phase UPLC on a Waters (Milford, MA) Acquity H-Class system equipped with a QDa single quadrupole mass spectrometer and an Acquity (Waters) 2.1 × 100 mm bonded ethyl hybrid (BEH) C_18_ column packed with 1.7 μm particles. The QDa was calibrated across the *m/*z range from 30 to 1,250 using the onboard calibrant. Mobile phase A consisted of water, phase B was 1% formic acid in water, and phase C was methanol. A flow rate of 0.5 ml min^−1^ was used and mobile phase B proportion remained at 10% throughout all gradients. Infusion experiments of individual polyphenol solutions into mobile phase flow to the QDa were used to determine optimal source and polarity settings for each compound, which were then assigned to either a positive or negative polarity panel. For each panel, selected ion recording channels corresponding to the optimal *m/z* value and determined source settings were established for each constituent polyphenol. Panels and settings information are described in Supplementary Tables S3, S4. The positive ion panel used a gradient of 5 to 30% C in 3.5 min, 30 to 60% C in 5.5 min, 60 to 90% C in 0.5 min, then a return to initial conditions in 0.5 min followed by a 4 min equilibration period. The negative panel utilized a gradient of 5 to 30% C in 3.5 min, 30–40% C in 1.9 min, 40–90% C in 0.35 min, then a return to initial conditions in 1 min, followed by a 3.75 min equilibration period. For the positive ion panel, a four-point standard curve was prepared with each point containing an equimolar mixture of all polyphenols across the range from 0.108 to 108 pmol μL^−1^. For the negative ion panel, a five-point standard curve was prepared with each point containing an equimolar mixture across the range from 0.100 to 286 pmol μL^−1^. 1 μl of all standard and bean seed coat and flower samples were injected.

At least 2 technical replicate injections were used to determine a mean concentration for all reported values. For samples with measured concentrations above the upper limit of quantitation, the corresponding sample was diluted to bring the measured concentration within range of the standard curve and the reported response for such values represents that determination multiplied by the dilution factor. Linear, non-weighted fits were used for all standard curves. The instrument control and quantitation functions were performed using Waters Empower 3 software.

### DNA Amplicon Sequencing

DNA was isolated from either leaf or embryo tissue using the Mag-Bind Plant DNA Plus kit (Omega Bio-Tek).^1^ The structure of the F3′5′H gene models from G19833 (*Phvul.006G018800*) and UI 111 (*PvUI111.06G022100*) were used to develop primers (Supplemenatry Table S5) for PCR amplifications. PCR fragments were amplified in a 25-μl volume using an amplification protocol with 45 cycles and annealing temperatures specific to each primer pair. DNA fragments were extracted with the NEB Monarch Gel Extraction Kit,^2^ and the fragments were Sanger sequenced by Eton Bioscience Inc.^3^

### Draft Genome Sequencing and Assembly

Thirty genotypes with various seed coat and flower color were selected for draft genome sequencing and assembly (Supplemenatry Table S1). For linked read sequencing, DNA was isolated from leaf tissue using the GE Healthcare Illustra DNA preparation kit (RPN8510). DNA was labeled with barcodes using the 10X Chromium Controller. The labeled DNA was sequenced on an Illumina HiSeq X Ten sequencer. A draft assembly for each genotype was generated from ~275,000,000 linked reads using the Supernova assembler with default settings (Weisenfeld et al., 2017).

### Protein Sequence Selection for F3′5H Analysis

For each species, the most recent reference genome represented in the Phytozome 13 database (accessed March 3, 2021)^4^ was searched with the key words “K13083” and “K05280,” the KEGG^5^ identifiers for F3′5′H and F3′H proteins, respectively. All protein sequences annotated with these identifiers were downloaded. Secondly, an exhaustive blastp search was performed using the *P. vulgaris* 5-593 F3′5′H protein sequence as a query at the NCBI protein database. Each Angiosperm order defined by APG IV (2016) was searched. To capture non-angiosperm sequences, all higher order taxa within the Viridiplantae^6^ were used as the database. All sequences identified by the blastp analysis were screened for the critical P450 heme-binding domain site (PFGAGRRICAG) and substrate recognition site 6 (SRS6). F3′5′H and flavonoid 3′ hydroxylase (F3′H) proteins vary at SRS6 position 8: F3′5′H = A or S; F3′5′H proteins = T (Seitz et al., 2007). Two F3′5′H Asterales proteins evolutionarily derived from F3′H genes (Seitz et al., 2015) were also included. The sequences were further limited to those without a deletion in either region of the other P450 essential domains and SRSs. Only 14 genes were excluded, and in each case, the excluded gene model was a duplicate of a gene with all functional domains in that species. Finally, all protein sequences were reannotated relative to KEGG nomenclature using the BlastKOALA web server (https://www.kegg.jp/blastkoala/). Only those sequences classified with the identifier “K13083” were used for the phylogeny analysis (Supplementary Table S6).

### Phylogenetic Network Development, Sequence Alignment, and Maximum Likelihood Phylogeny

A phylogenetic network was constructed using the median network approach with the nucleotide sequences for each *V* haplotype as implemented in SplitsTree (Huson and Bryan, 2006). The MUSCLE algorithm (Edgar, 2004) as implemented in the MEGA 7 package (Kumar et al., 2016) was used to align the full protein sequences. MEGA 7 was used to construct an unrooted 50% consensus maximum likelihood tree. The initial tree was defined by the Neighbor-Joining/BioNJ algorithms, and tree construction utilized the Jones–Taylor–Thornton substitution model. Evolutionary rates among sites were modeled as a Gamma distribution with five categories. The tree construction consisted of 500 bootstrap replicates.

### Protein Structure and Domain Discovery

The 3D structure of the 5-593F3′5′H protein was modeled using MODELLER (Webb and Sali, 2017), a comparative protein modeling program implemented at ModWeb.^7^ The transmembrane protein was predicted using the MINNOU method (Cao et al., 2006) available at http://minnou.cchmc.org/.

### Boxshade and WebLog Figure Development

Multiple sequence alignments were developed using the T-Coffee server,^8^ and boxshade displays were created using the BoxShade server.^9^ Critical CYP450 domains (Du et al., 2016) were extracted from the multiple sequence alignment. The WebLogo frequency bit scores (Crooks et al., 2004) were displayed graphically using the WebLogo WWW server.^10^

## Results

### Genetic, Physical, and Biochemical Mapping of *V* to the Heterochromatic Region of Pv06

**Figure 2:**
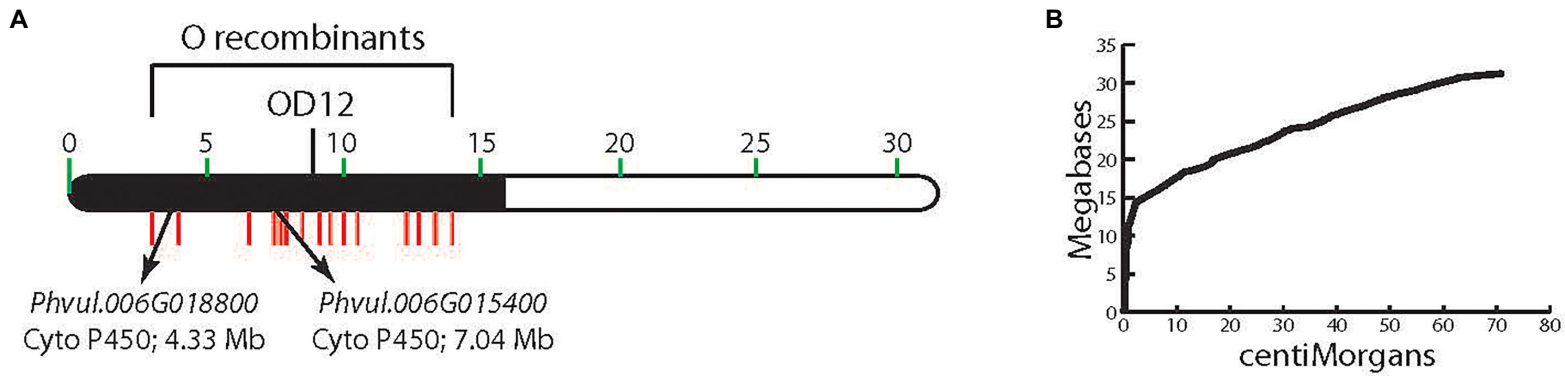
**(A)** The physical location of markers on chromosome Pv06 that cosegregate with *V*. **(B)** The megabase to centimorgan relationship for chromosome Pv06 showing the high Mb/cM ratio in the region to which *V* is mapped.

Genotypes with a dominant *V* allele are purple-flowered, and with a dominant *B* or dominant *G* and *B* alleles, express black-seeded beans. Homozygous recessive individuals have white (*v*) or pink (*v*^lae^) flowers, and their seeds lack dihydromyricetin-derived flavonoids. *V* was originally mapped to chromosome Pv06 in the 7–15 F_2_ population (McClean et al., 2002; Figure 2A). RAPD marker OD12_800_, located at position 9,288,093 bp in the extremely low recombination heterochromatic region of the chromosome, where the physical/genetic distance ratio is 2.1 Mb/cM (Figure 2B), co-segregated with *V*. Fifteen indel markers, located in the low recombination region, were polymorphic between the 7–15 parents (Moghaddam et al., 2014), and all co-segregated with *V* (Figure 2A). With such a low recombination rate in this region of Pv06, an alternative approach to fine-mapping *V* was necessary.

A detailed phytochemical analysis found that 5-593 (*V*) flowers and seeds contained myricetin 3-glucoside and delphinidin 3-glucoside (Table 2), two compounds that require F3′5′H activity. These compounds were absent from pink [PI 608680 (*v*^lae^)] and white-flowered [PI 608670 (*v*)] introgression lines, and greatly reduced or absent in the seed of those lines, respectively. With this background knowledge, a search for flavonoid pathway gene models within the low recombination Pv06 region of the reference Andean genome G19833 was undertaken. Two gene models (*Phvul.006G015400* and *Phvul.006G018800*; Figure 2A) with the F3′5′H KEGG identifier K13083, were found in the interval. The collective genetic and physical evidence that placed *V* in the low recombination region of Pv06 turned the focus to an analysis of natural variants of these two gene models in bean lines with different *V* alleles.

**Table 2:** Concentration of flavonoids in flowers and seed coats of genetic stocks carrying the *V*, *v*^lae^, and *v* alleles in 5–593 and introgression lines PI 608680 and PI 680679.

Flavonoid class	Flavonoid	Flowers (ηmol/g)			Seed coats (ηmol/g)		
		5–593 (*V*)	PI 608680 (*v*^lae^)	PI 608679 (*v*)	5–593 (*V*)	PI 608680 (*v*^lae^)	PI 608679 (*v*)
Anthocyanin	Delphinidin 3-glucoside	27.9	ND	ND	2,314.9	28.6	nd
Anthocyanin	Malvidin 3-glucoside	27.9	ND	ND	659.1	22.5	nd
Anthocyanin	Petunidin 3-glucoside	15.7	ND	4.3	795.1	23.4	2.9
Anthocyanidin	Delphinidin	+	+	+	+	ND	ND
Anthocyanidin	Malvidin	+	+	+	+	+	+
Anthocyanidin	Petunidin	+	+	+	+	+	+
Flavonol glucoside	Myricetin 3-glucoside	19.2	ND	ND	284.9	ND	ND
Flavonol	Myricetin	80.3	405.2	50.0	29.8	33.2	ND
Anthocyanin	Cyanidin 3-O-glucoside	ND	ND	ND	0.8	10.8	8.6
Anthocyanidin	Cyanidin	+	+	+	ND	+	ND
Flavonol glucoside	Quercetin 3-glucoside	ND	ND	ND	468.2	129.4	320.3
Flavonol	Quercetin	LLQ	ND	LLQ	737.8	1,078.2	1,089.4
Anthocyanin	Pelargonidin 3-glucoside	ND	ND	ND	ND	ND	ND
Anthocyanin	Pelargonidin	+	+	+	+	+	+
Flavonol glucoside	Kaempferol 3-sambubioside	11,322.3	52,560.3	8,443.5	nd	114.1	13.3
Flavonol glucoside	Kaempferol-3-O-glucoside	335.4	1,310.3	254.3	326.9	3,330.6	3,800.4
Flavonol	Kaempferol	5.2	17.2	6.5	9.2	151.8	214.8
Proanthocyanidins	Catechin	ND	ND	ND	252.8	580.4	636.9
Proanthocyanidins	Epicatechin	ND	ND	ND	ND	ND	ND
Proanthocyanidins	Procyanidin A2	ND	ND	ND	ND	ND	ND
Proanthocyanidins	Procyanidin B1	ND	ND	ND	250.5	484.3	638.8
Proanthocyanidins	Procyanidin B2	ND	ND	ND	ND	ND	ND
Proanthocyanidins	Cinnamtannin B1	ND	ND	ND	ND	ND	ND
Proanthocyanidins	Cinnamtannin A2	ND	ND	ND	ND	14.4	19.0

*ND = not detected; + = present but below lower limit of quantification*.

### Discovery of a *V* Gene Candidate Gene

Primers were designed to amplify each exon of these two models, and the protein structure was determined from the CDS sequence. The *Phvul.006G015400* amplicons from the two parents of the 7–15 F_2_ mapping population (McClean et al., 2002) were identical, and the eighth amino acid in the SRS6 domain was threonine which defines F3′H, not F3′5′H, function (Seitz et al., 2007). Therefore, *Phvul.006G015400* was excluded as a *V* candidate.

**Figure 3:**
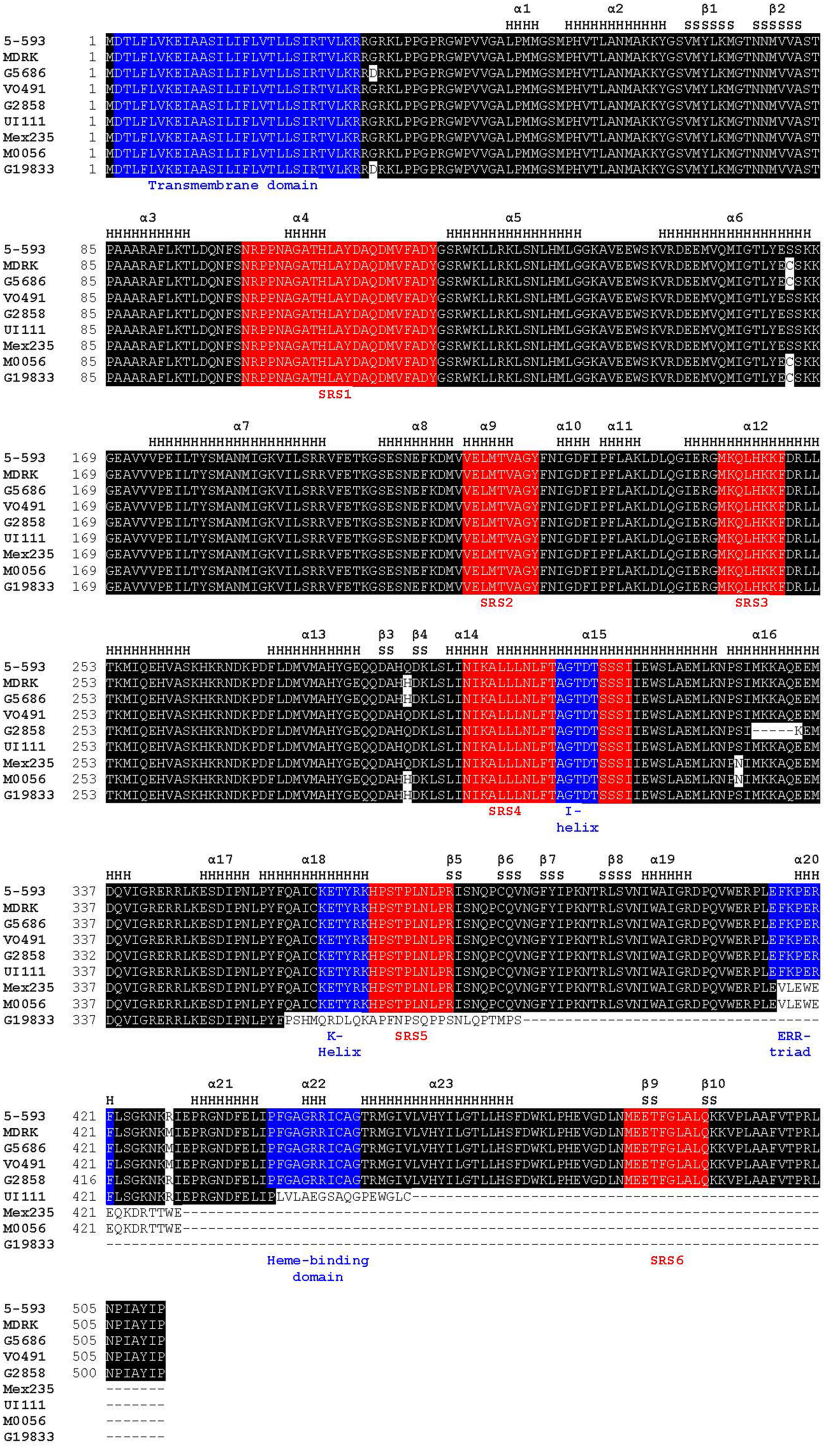
Amino acid alignment of proteins of representative genotypes for the nine *Phaseolus vulgaris* flavonoid 3′5′ hydroxylase haplotypes. Positions of α-helices (H) and β-sheets (S), as defined by MODELER, are noted above the alignment. The sequence substrate recognition sites highlighted in red, and CYP450 motifs shared among CYP450 proteins, highlighted in blue, are noted below the alignment. The following P450 regions were identified based on previous domain and matrix structure of the plant CYP75 family of proteins: SRS1, SRS2, SRS3, SRS4, I-helix (a subcomponent of SRS4), K-helix, SRS5, ERR-triad, heme-binding domain, and SRS6 (Falginella et al., 2010).

The 5-593 (*V*) *Phvul*.*006G018800* CDS was assembled from amplicon sequences, translated, and modeled as a member of the CYP450 protein family. While family members perform many biological functions, their sequences can be as little as 20% identical. Yet, their three-dimensional topology is highly conserved (Graham and Peterson, 1999). The topology consists of two neighboring α-helices clusters that interact to form a structural core to which the heme binds, and β-sheets 1 and 2 that provide the substrate access point. Four conserved motifs (I-helix, K-helix, ERR-triad, and heme-binding) are associated with these structural domains. Six substrate recognition sites (SRS1-6) are nearly universally found in CYP450 proteins and are important for substrate binding and the enzymatic reaction (Gotoh, 1992). Sequence analysis of the 5-593 *Phvul*.*006G018800* protein identified the six conserved substrate recognition sites (SRS) and the four functional motifs typical of CYP enzymes (Figure 3). These domain sequences, except for the transmembrane domain, were nearly identical to those found in functional F3′5′Hs of other legumes (Supplemenatry Figure S1). The CDS from three black-seeded, purple-flowered Andean genotypes (Black Wonder, RH No. 6, W6 9644; Table 1) were identical to the Middle American 5-593.

The CDS sequences of *Phvul*.*006G018800* from 5-593 and the reference G19833 differed by seven SNPs and a single cytosine insertion. The G19833 insertion led to sequence divergence of 28 amino acids beginning at amino acid 358, and an early stop codon that truncated the protein by 125 amino acids. This truncation eliminated the k-helix, SRS5, ERR-triad, heme-binding domain, and SRS6 (Figure 3). The *Phvul.006G018800* sequences for multiple homozygous dominant purple-flowered plants of the 7–15 population were identical to 5-593, while the white-flowered plants sequence was identical to the ICA-Calima sequence. The *Phvul.006G018800* sequence variation between 5-593 and G19833 was further associated with *V* by genetically testing three polymorphic KASP markers located in the coding region (Supplementary Table S7) on a F_2_ population developed by crossing the purple-flowered Black Magic parent, whose *Phvul.006G018000* sequence is identical to 5-593, and white-flowered introgression line *g v* BC_3_-5-593. All three markers co-segregated with flower color.

### Natural Variants of the *V* Candidate Gene

*Phvul.006G018800* was next sequenced from V0491 (PI 527745) and *v*^lae^ BC_3_ 5-593, two genotypes with pink flowers and mineral brown seeds. Their sequence differed from 5-593 by two SNPs and one amino acid substitution (Figure 3). The R428M amino acid substitution is located near the critical PERF motif that stabilizes the heme in P450 enzymes (Du et al., 2016). M0056, the *v* donor for the *v* BC_3_-593 introgression line, with mineral brown seed coats and pink flowers, differed from 5-593 by one SNP and a 14-nucleotide deletion in exon three that changed 14 amino acids beginning at amino acid 416 followed by the introduction of a frameshift stop codon that resulted in the deletion of the terminal 97 amino acids (Figure 3, Supplementary Table S8). The deletion altered the ERR-triad and deleted the heme-binding domain and SRS6.

A blastp analysis identified *PvUI111.06G022100* as an ortholog of *Phvul.006G018800* in the high quality, long-read race Durango pinto UI 111 common bean reference assembly.^11^ This pinto genotype has white flowers and a brown mottle seed coat pattern on a cartridge buff background and was previously determined to carry the recessive *v* allele (Prakken, 1977). A nucleotide (T) deletion at position 1,323 relative to 5-593 was detected in the UI 111 CDS that altered the protein sequence starting at amino acid position 441 and introduced an early stop codon 16 amino acids further downstream that eliminated the essential heme-binding domain and SRS6 (Figure 3). Most recently, Labor Ovalle, a race Guatemala landrace with black seeds and purple flower was sequenced with long-read technology.^12^ Gene model *PvLabOv.06G020400* was found to be identical to the 5-593 protein sequence. This result reiterates the close relationship between the Guatemala and Mesoamerica common bean races (Tobar Pinon et al., 2021).

Draft genome assemblies were developed for 30 *P. vulgaris* genotypes, with varying flower and seed coat phenotypes, by sequencing 10X linked read libraries and assembling scaffolds. The assemblies ranged from 470 Mb to 570 Mb in size, with an average of 523 Mb. The contig N50 ranged from 26 Kb to 88 Kb with an average of 70.29 Kb. The scaffold N50 ranged from 32 Kb to 4.5 Mb with an average of 1.1 Mb (Supplementary Table S1). Each genotype with white flowers and seeds was homozygous for a recessive *p* allele. The gene model associated with the *V* gene was determined for each genotype by homology to the G19833 *Phvul.006G018800* and UI 111 *PvUI111.06G022100* gene models. Four additional alleles were identified. White-flowered Mex235 contained a 14 nt deletion, white-flowered G2858 contained a 15 nt deletion, pink-flowered MDRK contained an additional four nucleotide insertion relative to V0491, and pink-flowered G5686 had an additional SNP relative to MDRK. Among all genotypes screened, a total of nine *P*. *vulgaris* F3′5′H CDS haplotypes were discovered (Figure 3, Supplementary Table S8), and the alleles were annotated with a superscript designation of the reference genotype.

### Phylogenetic Network, Intragenic *V* Gene Recombinants, and Evidence That *V* Encodes F3′5′H

**Figure 4:**
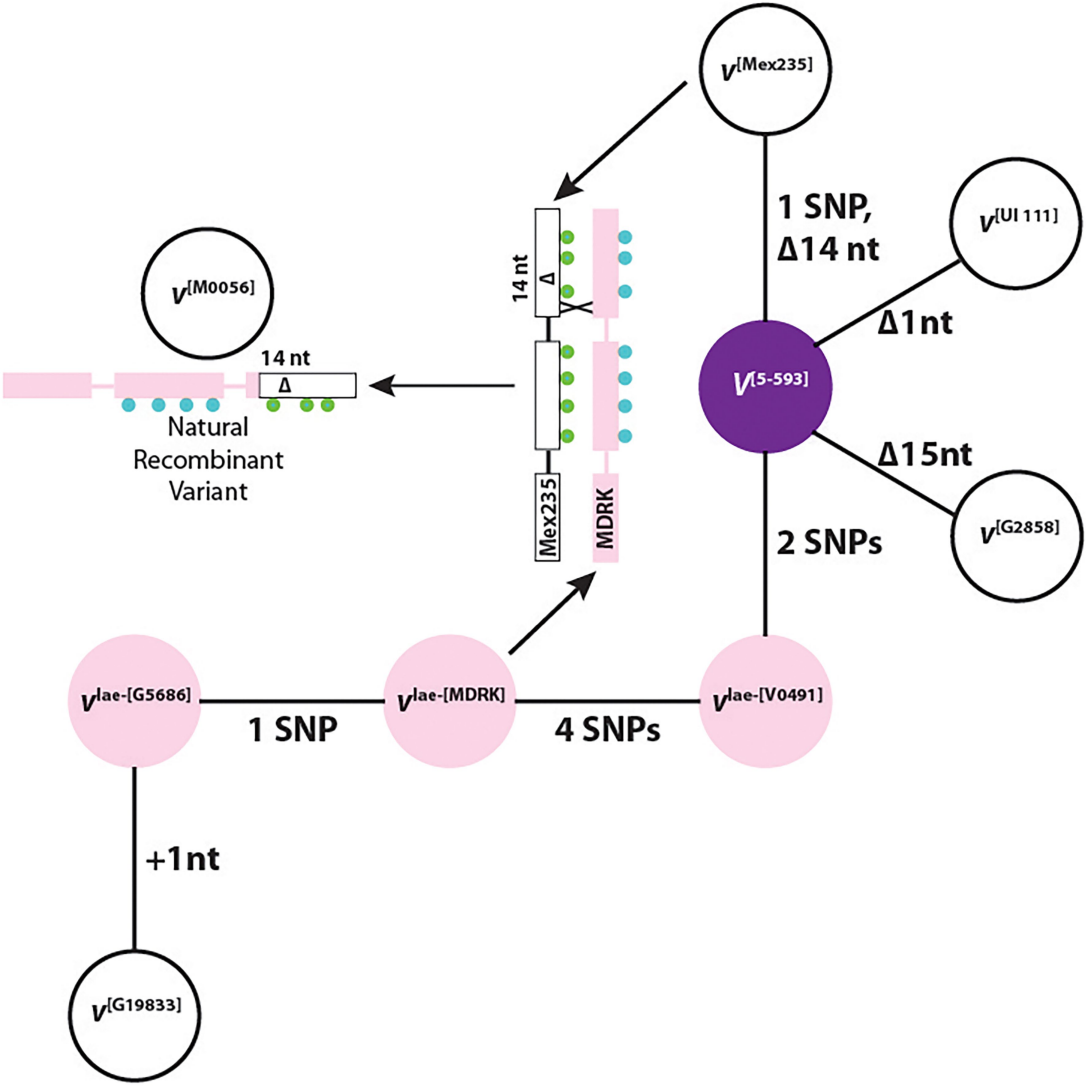
A haplotype phylogenetic network of dominant (*V*) and recessive (*v*, *v*^lae^) alleles. The colors represent the flower color of each genotype. The representative genotype for each haplotype is in bracketed superscript. +, a nucleotide insertion; Δ, a nucleotide deletion. The green and blue circles represent the SNP differences between the *v*^[Mex235]^ and *v*^lae-[MDRK]^ haplotypes, respectively. The number preceding “SNP” indicates the number of single nucleotide polymorphism (SNP) differences between neighboring haplotypes.

A phylogenetic network shows a direct mutational relationship between all haplotypes with the dominant V^[5-593]^ as the hub haplotype (Figure 4). This is expected since this haplotype is shared by the two gene pools. Three recessive white-flowered haplotypes were derived directly from *V*^[5-593]^. The *v*^lae-[V0491]^ haplotype links the Middle America gene pool to the Andean gene pool haplotypes *v*^lae-[MDRK]^, *v*^lae-[G5686]^, and *v*^[G19833]^ through SNPs and a single nucleotide insertion. Nucleotide and amino variants for each haplotype are found in Supplementary Table S8.

The phylogenetic network revealed an important relationship between the F3′5′H coding region of the *v*^[Mex235]^, *v*^lae-[MDRK]^, and *v*^[M0056]^ haplotypes which provides evidence that *V* indeed encodes F3′5′H. The explanation begins with the F_2_ population from the cross of 5-593 (*V*^[5-593]^ *sal*) and M0056 (*v*^[M0056]^ *Sal*) described previously (Bassett et al., 1990). In this population, individuals homozygous recessive for *Sal* (dominant *Sal* is epistatic to all *V* alleles), segregated 3:1, purple to white flowers. Therefore, for white flowers to appear, M0056 must possess a non-functional *v* gene. When the F3′5′H coding regions for the *v*^[Mex235]^, *v*^lae-[MDRK]^, and *v*^[M0056]^ haplotypes are aligned, an intragenic crossover event is observed between the *v*^lae-[MDRK]^ and *v*^[Mex235]^ haplotypes that generated the *v*^[M0056]^ haplotype (Figure 4). This natural recombinant haplotype contains the 5′ end of the *v*^lae-[MDRK]^ haplotype, and the 3′ end of the *v*^[Mex235]^ haplotype with the 14 nt deletion that eliminates the heme-binding domain and SRS6. Therefore, a natural common bean recombinant variant, generated by an intragenic crossover event between a chromosome that expressed white flowers and a chromosome that expressed pink flowers, contains a deletion that eliminated the F3′5′H function of the pink haplotype. This is equivalent to gene editing, where if a portion of a candidate gene is deleted and a phenotypic change occurs, it is concluded that the candidate gene is indeed the gene of interest. Therefore, the *V* gene encodes the F3′5′H protein and provides the important function of enabling the production of dihydromyricetin-derived flavonoids.

### Orthologs of *V* Control Flower Color in Other Legumes

*V* orthologs in other legumes were discovered by a blastp search. The classic soybean (*Glycine max* L.) *W1* flower color gene, that imparts purple color and encodes a F3′5′H (Zabala and Vodkin, 2007), was the top ortholog. The white flower *w1* allele contains a tandem repeat that introduces an early stop codon that eliminates the critical C-terminal SRS6 domain. The light purple flowers of the *G. soja w1-lp* allele have greatly reduced levels of dihydromyricetin-derived flavonoid glucosides (Takahashi et al., 2010). A V210M substitution in the third amino acid of SRS2 in the w1-lp protein occurs in a residue that is invariant among all legume F3′5′H proteins that produce purple flowers. The *B* locus in pea (*Pisum sativum* L.) encodes F3′5′H (Moreau et al., 2012), and the recessive pink-flowered *b* allele contains a 23 nt deletion that introduces a premature stop codon that eliminates part of SRS1 and all other functional domains. The mutant flowers lack delphinidin and petunidin derivatives found in the wild-type genotype. A single nucleotide change was detected in a second pink-flowered *b* mutant that resulted in a single amino acid change (G → E) of glycine residue five in SRS1 that is invariant in all legume F3′5′H proteins (Supplementary Figure S1). The importance of this amino acid was further demonstrated in sweet pea (*Lathyrus odoratus*) where a pink-flowered mutant of the classic *A1* gene (Punnett, 1923), which also encodes F3′5′H (Xue and Cronk, 2017), contains the same G → E SRS1 substitution observed in the *P. sativum* mutant. Finally, the *P. vulgaris* 5-593 F3′5′H protein and its ortholog in the black-seeded tepary bean (*P. acutifolius A. Gray*; Moghaddam et al., 2021) were 96% identical with only a single amino acid substitution in the SRS1 domain (Supplementary Figure S1).

### Domain/Motif Analysis of F3′5′H in Land Plants

**Figure 5:**
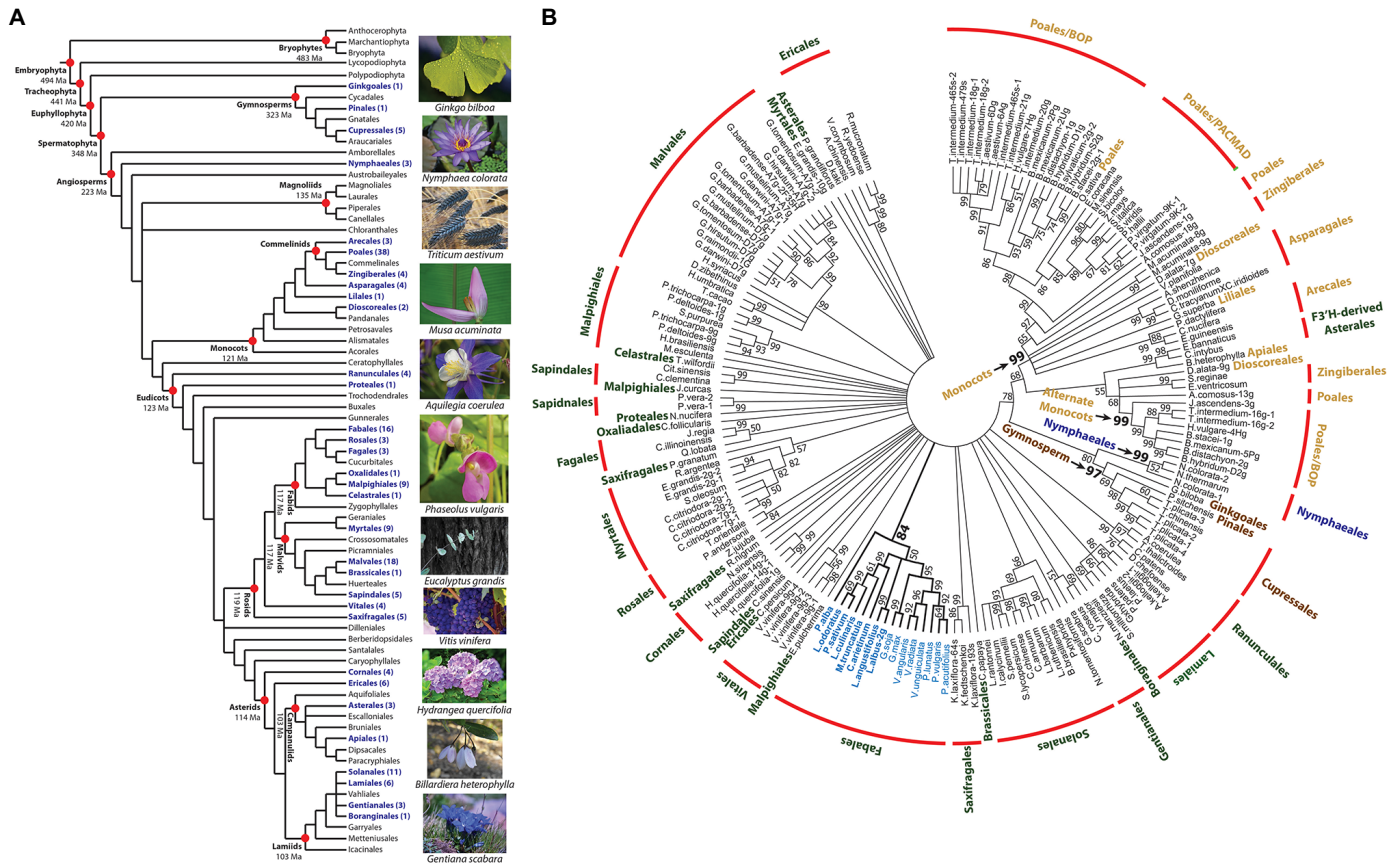
Phylogenetic distribution and sequence relationships for land plant flavonoid 3′5′ hydroxylase proteins. **(A)** Distribution of F3′5′H proteins among land plants higher order taxa. The tree was assembled by augmenting the 2016 angiosperm phylogeny (APG IV, 2016) with the evolutionary dating data in Morris et al. (2018), and gymnosperm ordering in Forest et al. (2018). The dating of the orders in the eudicots was based on Magallón et al. (2015). All other datings were based on the results (mean of range) for the monophyletic model presented in Table 3 of Morris et al. (2018). All taxa containing a species with a F3′5′H protein sequence are noted in purple. **(B)** Unrooted 50-percent consensus maximum likelihood tree of land plant F3′5′H protein sequences. The colors for each order are: water lily = blue; gymnosperms = brown; monocot = tan; and eudicot = green. Those nodes with bootstrap values >50% are noted.

To assess the distribution, structural variation, and phylogeny of F3′5′H across the breadth of land plants, *V* gene orthologs were mined from the Phytozome and NCBI genome databases. The protein sequences were selected based on functional features repeatedly shown to be necessary for the F3′5′H enzymatic reaction (Graham and Peterson, 1999). This whole-genome, computational approach, based on domains experimentally proven to be critical to the function of a gene, are preferred to simple homology searches (Restrepo-Montoya et al., 2020). This exhaustive search identified 177 F3′5′H protein coding sequences from 129 land plant species. The gene was not detected in Chlorophyta. Within the Embryophyta, the gene was not detected in ferns or bryophytes. The presence of F3′5′H in the gymnosperms (Figure 5A) dates the gene back to the appearance of Spermatophyta (348 Ma; Morris et al., 2018). The F3′5′H gene is found in the Nymphaeales basal angiosperms and sporadically throughout other angiosperm orders. The gene was absent in the Magnoliales. A search of the 129 species with a whole-genome reference assembly and annotation in the Phytozome database that represents a broad taxonomic collection of the Viridiplantae, found only 53 species contained a F3′5′H gene model with all essential domains. The gene was discovered in all monocot species and nine of the ten Fabales species (except peanut, *Arachis hypogaea*). It was absent in all 28 Phytozome members of the Brassicaceae family. When the NCBI and Phytozome sequences were combined, F3′5′H was found in the genome of 29 of the 64 Angiosperm orders (APG IV, 2016) and three of the six Gymnosperm orders (Figure 5A; Forest et al., 2018).

**Figure 6:**
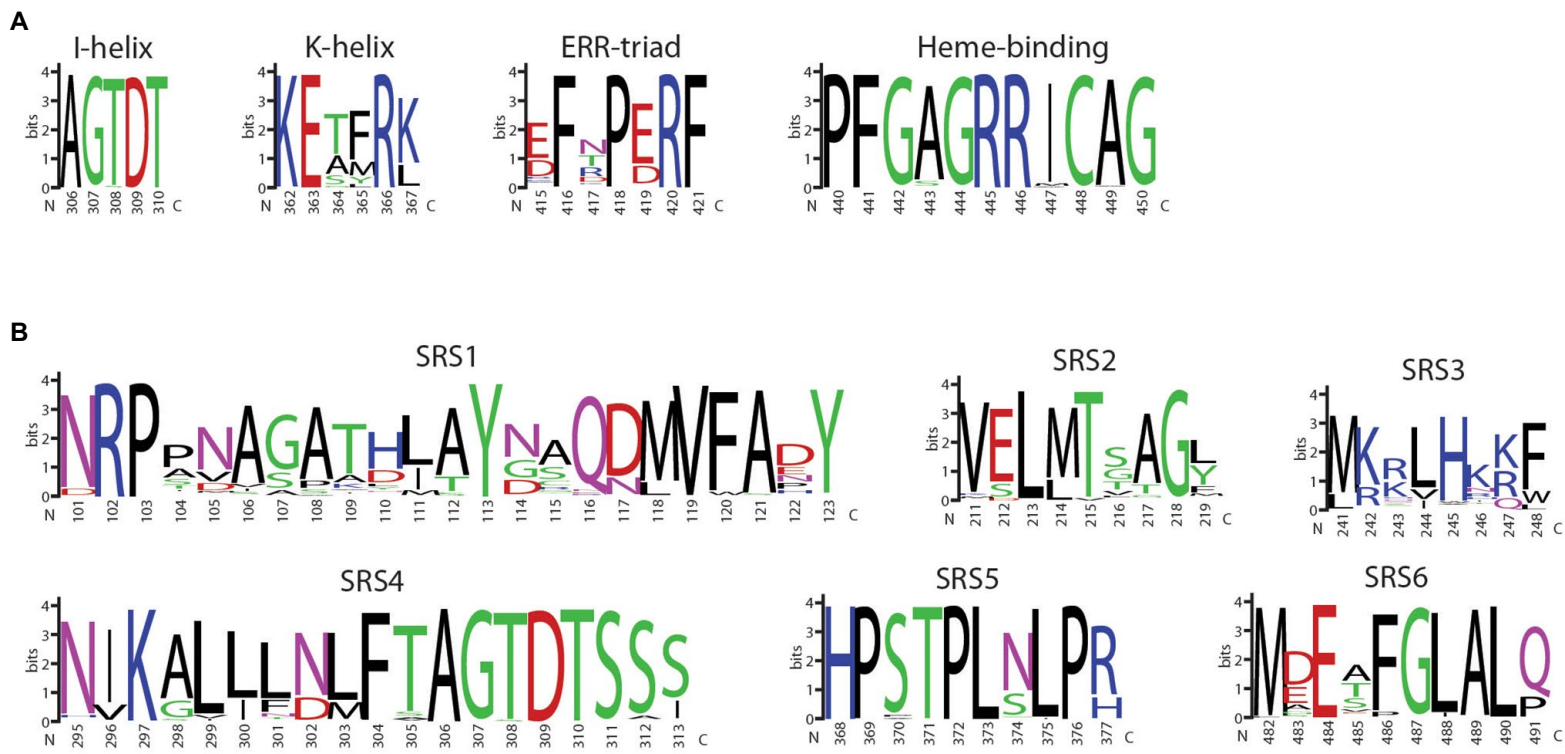
WebLogo display of the **(A)** four core CYP450 motifs and **(B)** six sequence recognition sequences (SRS) of land plant flavonoid 3′5′ hydroxylase proteins. The logos are based on the multiple sequence alignment using MUSCLE of 177 land plant F3′5′H protein sequences identified from an exhaustive search of Phytozome and NCBI protein databases. The amino acid positions are based on the sequence of 5-593 of *P. vulgaris*.

The 177 F3′5′H proteins were aligned, and the highly conserved CYP450 motifs and SRSs were visualized (Figures 6A,B). SRS1 is the most variable region with 86 unique sequences (Figure 6B). The amino acids 245H, in SRS3, and 302N in SRS4, previously shown to be under positive selection (Jia et al., 2020), were nearly invariant among all species. SRS4, which extends across helix I and is an important component of the functional pocket of P450s and associated with oxygen binding, is well-conserved. Among all species, the SRS4 I-helix motif was invariant. Only 10 SRS5 sequences and 21K-helix motif variants were observed. This conservation is not unexpected since amino acid variants in these two adjacent features can alter P450 hydroxylation (Richardson and Johnson, 1994) and stereoselectivity in humans (Ellis et al., 1996), as well as substrate binding orientation in plants (Schalk and Croteau, 2000). Similarly, a single substitution, Ala/Ser vs. Thr, at amino acid position nine in SRS6, defines the 3′5′ versus 3′ hydroxylation of dihydrokaempferol (DHK), respectively (Seitz et al., 2007).

### Phylogeny of the Land Plant F3′5′H Gene Family

A 50% consensus maximum likelihood gene tree was constructed with the full F3′5′H protein sequence collection (Figure 5B). A single clade with a bootstrap support value (BSV) = 78 revealed a relationship between Gymnosperms, Nymphaeles, and a subset of Poales proteins. Within this clade, strongly supported subclades for each phylogenetic group were detected with BSV values of 97, 99, and 99, respectively. Unlike this group of land plants, a single eudicot clade was not detected. Rather, all species in multiple orders (Cornales, Fabales, Fagales, Gentianales, Lamiales, Malvales, Rannuncales, Rosales, and Solanales) formed a clade. For the Fabales species, which are all members of the Papilionoideae subfamily, the F3′5′H gene tree is consistent with a genus-level legume species tree based on multilocus sequence data (Koenen et al., 2020).

The F3′5′H duplication history was considered for the 25 Phytozome reference genomes assemblies which have multiple copies of the gene. Duplicated copies could be traced to polyploidization events, such as with switchgrass (*Panicum virgatum*), where duplicates were located on chromosome 9 of the K and N subgenomes (Lovell et al., 2021). With *Eucalyptus grandi*s, the duplicates on chromosomes 2 and 10 were the result of the whole-genome duplication, while an additional copy on chromosome 2 appeared *via* tandem duplication. These types of duplication events are representative of many *E. grandi*s genes (Myburg et al., 2014). Interpreting the duplications found in the Poales is more challenging. Except for *Panicum virgatum*, as noted above, all Poales/PACMAD clade species only contain a single F3′5′H gene copy. By contrast, all Poales/BOP species contain multiple copies. These copies were found in two well-supported clades, Poales/BOP I and II (Figure 5B). The copies in Poales/BOP II, the smaller of the two clades, are not on the same chromosome as those genes found in Poales/BOP, suggesting a different evolutionary arc for these genes.

## Discussion

Common bean is recognized for its variety of seed coat and flower colors and patterns. At least 13 genes (*T*, *P*, *C*, *R*, *J*, *G*, *B*, *V*, *Rk*, *Gy*, *Sal*, *Z*, *Bip*) regulate color expression, and many of these genes are presumed to have a regulatory or enzymatic function in the flavonoid pathway. This was the case for *P*, which encodes an ortholog of the βHLH transcription factor that is a component of the MBW complex which regulates the late enzymes of the pathway (McClean et al., 2018). Feenstra (1960) suggested *V* performed an enzymatic function when, based on biochemical evidence, he hypothesized that *V* encodes F3′5′H. Here, we have shown that *V* indeed encodes F3′5′H. The definitive proof was the discovery of the *v*^lae[M0056]^ haplotype that resulted from a natural intragenic recombination event between the *v*^lae-[MDRK]^ (pink flower) and *v*^[Mex235]^ (white flower) haplotypes that introduced a deletion to the *v*^lae-[MDRK]^ haplotype that resulted in a frame shift stop codon which eliminated the essential heme-binding and SRS6 regions. This event converted a “pink” haplotype into a “white” haplotype. The recombination event is a natural equivalent to gene editing. When gene editing technology is used to delete a portion of a candidate gene, and the edited progeny shows a phenotypic change, the candidate gene is proven to be the causative gene for the phenotype of interest. For a species, such as *P. vulgaris*, where gene editing and other complementation techniques are not available, a sequence analysis of large numbers of natural variants available in seed banks is one alternative to providing the definitive proof necessary for successful gene cloning experiments.

The chemical analysis of flowers and seed coats of the *V*, *v*^lae^, and *v* genotypes provided insight into the function of the flavonoid pathway in different tissues. For *V* genotypes, dihydromyricetin-related compounds, which require the action of F3′5′H, were detected in both tissues, but to a much greater extent in seed coats. The appearance of reduced levels of dihydromyricetin derivates in *v*^lae^ seed coats, but not flowers, suggests a regulatory mutation in *v*^lae^ suppresses expression of the allele in flowers to a greater extent than seed coats. Scanning ~3,000 nt upstream region of the ATG start site in several *v*^lae^ draft genomes revealed large deletions that may contain sequences that regulate the quantitative or spatial expression of F3′5′H. These deletions should be investigated to understand the structure/function relationship with regards to the differential expression. Dihydroquercetin-related compounds, which require F3′H for synthesis, were found in seed coats but not flowers of any of the three genotypes. This suggests F3′H expression is differentially regulated between the two tissues. Similarly, only two classes of proanthocyanidins were observed in seed coats and none were present in flowers. Collectively, the chemical analysis of the introgression lines demonstrates tissue-specific expression at several steps along the flavonoid pathway.

The evolutionary trajectories of the flavonoid pathway genes in the Middle American and Andean gene pools appear to differ. A dominant ancestral haplotype, *V*^[5-593]^, controls purple flower color in both the Middle American and Andean gene pools. This suggests the haplotype existed prior to the gene pool divergence ~113 kya (Mamidi et al., 2013). This contrasts with the flavonoid pathway regulator gene *P* (McClean et al., 2018), the growth habit *Fin* gene (Kwak et al., 2012), and the photoperiod sensitivity gene *Ppd* (Weller et al., 2019), where gene pool-specific dominant alleles evolved first and subsequent recessive alleles in each gene pool appeared later. Collectively, eight recessive *V* haplotypes that control pink or white flower color evolved from *V*^[5-593]^ evolved in the two gene pools. The early appearance of the white *v*^[Mex235]^ haplotype is supported by the discovery of this haplotype in both gene pools. The other haplotypes were gene pool-specific. The discovery of the Andean *v*^lae^ haplotypes supports the almost exclusive appearance of pink flowers in that gene pool.

In the Middle American gene pool, flowers are purple or white. The mutations in the two Middle American *v* alleles occurred in regions necessary for F3′5′H function. The fact that mutations result in white flowers suggests other flavonoid pathway genes were not functioning in Middle American flowers. In contrast, while the vast majority of Andean F3′5′H mutations are also in functional domains, those genotypes most often express pink flower color. Therefore, genes necessary for the production of dihydroquercetin and its derivatives are functioning properly in flowers of Andean genotypes with the *v*^lae^ allele. From a phenotypic perspective, the evolution of the pathway in the Andean gene pool appears consistent with pea (Moreau et al., 2012), petunia (Matsubara et al., 2005), and gentian (Nakatsuka et al., 2006) where a mutation in functional domains of the F3′5′H protein resulted in pink flowers. Collectively, these results suggest that as with other plant species, the bean flavonoid pathway genes follow unique evolutionary arcs in the two gene pool. This supports the utility of *Phaseolus* species as useful models to study replicated evolution in a single species (Debouck, 1996; Gaut, 2014; Bitocchi et al., 2017; Cortés et al., 2018).

In theory, mutations in any gene encoding an enzyme or transcription factor necessary for the synthesis of flavonoids can shift color expression in flowers and/or seeds. F3′5′H along with F3′H, DFR, and FLS function at a branch point in the pathway, and their competition for the DHK substrate largely determines the color that will be expressed. While a mutation of each of these genes has been responsible for color evolution in some plant species, most color transitions involved mutations in F3′5′H and to a lesser extent F3′H (Wheeler and Smith, 2019). What might be the reason for the enriched number of F3′5′H mutations in plants relative to the other targets genes in the pathway? One possibility is that F3′5H has limited pleiotropic effects on other critical phenotypes, and mutations in the gene do not drastically affect fitness. Conversely, other pathway genes may have pleiotropic effects on multiple phenotypes necessary for normal growth and development, and mutations in those genes would reduce fitness.

We addressed this from a phylogenetic perspective by performing a domain-based search of the complete set of Phytozome genome assemblies to assess the distribution of F3′5′H and F3′H. Only 41% of the genomes contain a F3′5′H gene with a complete set of functional domains. By contrast, F3′H was found in nearly all Angiosperm genomes for which a full reference assembly is available. This suggests that F3′H and its quercetin and cyanidin flavonoids products may have important roles in multiple phenotypes critical to the growth and development of plants. Indeed, the importance of F3′H was confirmed in Arabidopsis when it was shown that *tt7* mutants, that lack a functional F3′H gene, had suboptimal growth under UV-B stress (Ryan et al., 2001). Additionally, wild-type Arabidopsis does not have a F3′5′H gene yet grows normally under UV-B stress. This suggests that myricetin-derived flavonoids are not necessary to combat the stress (Li et al., 1993). The observation that cyanidin is found in vegetative tissues of ~90% of plant species, whereas pelargonidin and delphinidin are rarely found in vegetative tissues (Wessinger and Rausher, 2012) further emphasizes the importance of F3′H for plant growth and development since it is required for the synthesis of cyanidin. Indeed, it has been demonstrated that dihydroxylated flavonols products of F3′H enzymatic activity provide greater protection against UV-B (Agati et al., 2013), salinity (Agati et al., 2011), and drought stresses (Nakabayashi et al., 2014) than other flavonols. Assuming the relationship between dihydroxylated flavonoids and abiotic stress tolerance is widespread in plants, our phylogenetic survey supports the suggestion of Wessinger and Rausher (2012) that the abiotic stress protection provided by dihydroxylated flavonoids favored the evolutionary maintenance of a functional F3′H gene in nearly all land plant species. Conversely, without such a significant pleiotropic effect on abiotic stress, or other important growth and development phenotypes, mutations in regulatory or functional domains of F3′5′H genes could lead to the wide array of white, pink, blue, and purple flower colors seen in beans and throughout the Angiosperms.

## Data Availability Statement

The datasets presented in this study can be found in online repositories. The names of the repository/repositories and accession number(s) can be found in the article/Supplementary Material.

## Author Contributions

PM, RL, JO, and PM designed the project. RL, AH, RS, TT, and MZ designed the amplicon sequencing protocol and completed that sequencing. RL and CO completed the F2 mapping experiment and designed the KASP markers. RG, KH, and TT designed and completed the flavonoid analysis. JS, JG, SL, CP, and MR created the 10X linked read libraries and completed their sequencing and scaffold assembly. PM performed the phylogenetic analyses. All authors participated in writing and/or editing the manuscript.

## Funding

Funding was provided by the USDA, Agricultural Research Service through the Pulse Crop Health Initiative, Agreement no. 58-3060-0-041. We thank the following researchers for providing early access to annotation data from individual reference genomes prior to publication: Joerg Bohlmann, *Thuja plicata* (v3.1); Jorge Alexander Duitama Castellanos, *Phaseouls lunatus* (v1); Tim Close, *Vigna unguiculata* (v1.2); Dave Des Marais, *Brachypodium mexicanum* (v1.1); Katrien Devos, *Eleusine coracana* (v1.1); Robert Henry, *Corymbia citriodora* (v2.1); Don Livingston, *Theobroma cacao* (v2.1); Henry Nguyen, *Glycine soja* (v1.1); Jennifer Randall, *Carya illinoinensis* (v1.1); John Vogel, *Brachypodium distachyon* (v3.2) and *Brachypodium sylvaticum* (v1.1); and Xiaohan Yang, *Kalanchoe laxiflora* (v1.1).

## Conflict of Interest

The authors declare that the research was conducted in the absence of any commercial or financial relationships that could be construed as a potential conflict of interest.

## Publisher’s Note

All claims expressed in this article are solely those of the authors and do not necessarily represent those of their affiliated organizations, or those of the publisher, the editors and the reviewers. Any product that may be evaluated in this article, or claim that may be made by its manufacturer, is not guaranteed or endorsed by the publisher.

## Footnotes

^1^ https://www.omegabiotek.com/product/mag-bind-plant-dna-plus-96-kit/

^2^ https://www.neb.com/products/t1020-monarch-dna-gel-extraction-kit

^3^ https://www.etonbio.com/

^4^ https://phytozome-next.jgi.doe.gov/

^5^ https://www.genome.jp/kegg/

^6^ https://www.ncbi.nlm.nih.gov/Taxonomy/Browser/wwwtax.cgi?id=33090

^7^ https://modbase.compbio.ucsf.edu/modweb/

^8^ http://tcoffee.crg.cat/apps/tcoffee/do:regular

^9^ https://embnet.vital-it.ch/software/BOX_form.html

^10^ https://weblogo.berkeley.edu/logo.cgi

^11^ https://phytozome-next.jgi.doe.gov/info/PvulgarisUI111_v1_1

^12^ https://phytozome-next.jgi.doe.gov/info/PvulgarisLaborOvalle_v1_1

## Supplementary Material

Supplementary Figure S1 Amino acid alignment of flavonoid 3′5′ hydroxylase proteins for eleven legume species. The sequence divergence between species in the Indigoferoid (common bean, etc.) and Hologalegina (chickpea, etc.) crown nodes [as defined by Lavin et al. (2005)] precluded the discovery of a definitive transmembrane domain. The sequence substrate recognition sites are highlighted in red and CYP450 motifs shared among CYP450 proteins are highlighted in blue. The gene source is listed in Supplementary Table S6.
