# Supplementary figures and images for: The Common Bean V Gene Encodes Flavonoid 3′5′ Hydroxylase: A Major Mutational Target for Flavonoid Diversity in Angiosperms
Source: Front Plant Sci. 2022 Mar 31;13:869582. doi: 10.3389/fpls.2022.869582 (PMC9009181; doi:10.3389/fpls.2022.869582)

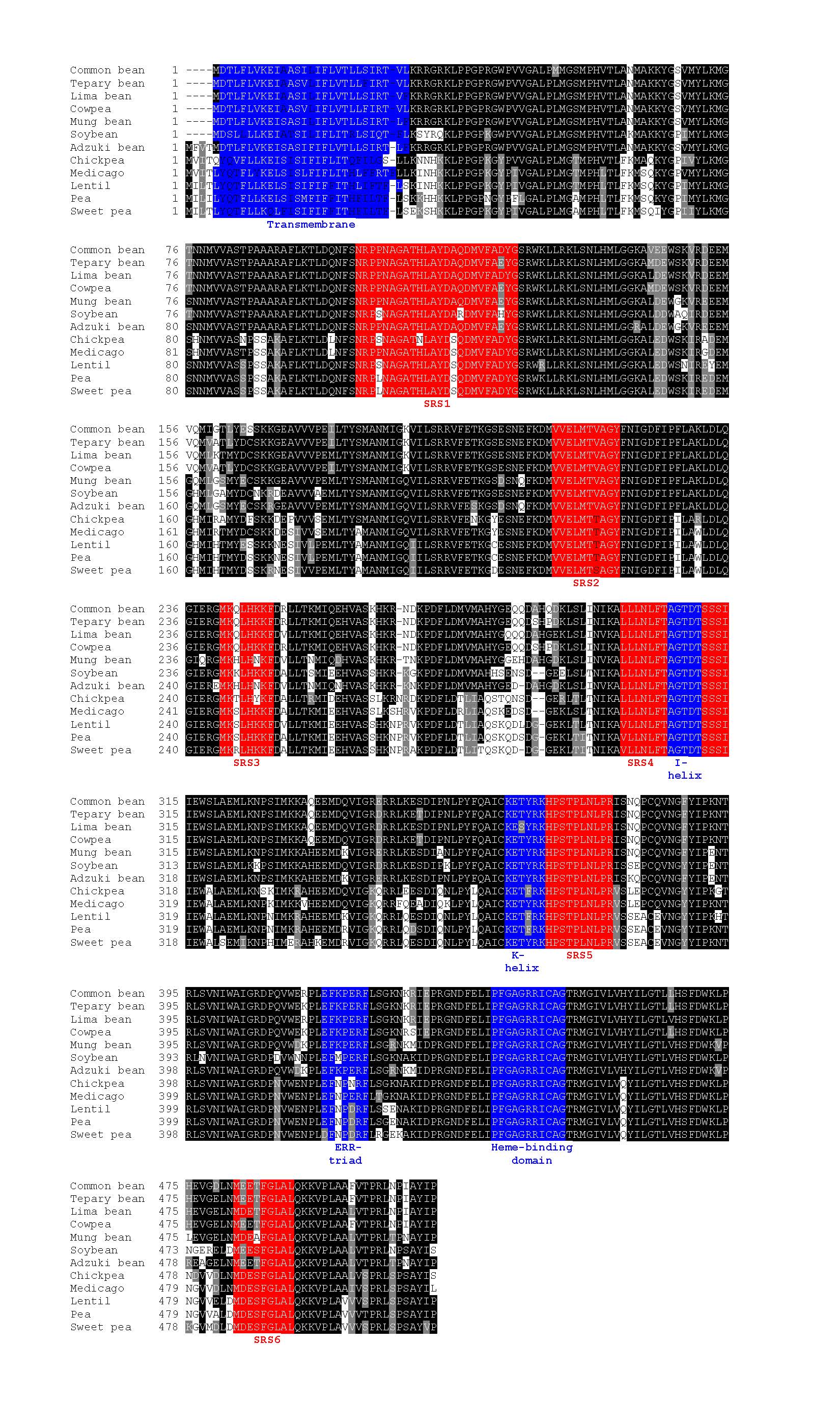

Supplement: Supplementary Figure S1 — Amino acid alignment of flavonoid 3′5′ hydroxylase proteins for eleven legume species. The sequence divergence between species in the Indigoferoid (common bean, etc.) and Hologalegina (chickpea, etc.) crown nodes [as defined by Lavin et al. (2005)] precluded the discovery of a definitive transmembrane domain. The sequence substrate recognition sites are highlighted in red and CYP450 motifs shared among CYP450 proteins are highlighted in blue. The gene source is listed in Supplementary Table S6. [file Image_1.JPEG]
